# Supplementary material for: CELA3B immunostaining is a highly specific marker for acinar cell carcinoma of the pancreas
Source: PLoS One. 2023 Jun 28;18(6):e0287528. doi: 10.1371/journal.pone.0287528 (PMC10306197; doi:10.1371/journal.pone.0287528)
Supplement: Attachment — Submitted filename: ReplyLetter_CELA3B_141222.docx [file pone.0287528.s001.docx]

|  |  |  |  |  |
| --- | --- | --- | --- | --- |
|  | **Institut für Pathologie**  Prof. Dr. Ronald Simon |  | Martinistraße 52  20246 Hamburg  Telefon: (040) 7410-57214  Telefax: (040) 7410-55997  r.simon@uke.de  www.uke.uni-hamburg.de |  |
|  |  |  |  |  |
| Emily Chenette, PhD  Editor-in-Chief PLOS One |  | | December 14, 22 |  |

Dear Reviewers and dear Prof Chenette,

Please find enclosed our revised manuscript entitled “**CELA3B complements CPA1 in the diagnosis of acinar cell carcinoma of the pancreas**”. We would like to thank the reviewers for their comments on how to improve the manuscript. Please find below our point-by-point responses to the academic editor and the reviewers.

**In reply to the Academic Editor**

When submitting your revision, we need you to address these additional requirements.

1. Please ensure that your manuscript meets PLOS ONE's style requirements, including those for file naming.

**Reply: We have now the update the PLOS ONE's style requirements in our manuscript.**

2. If you would like your manuscript to be considered for this collection (Early Detection, Screening and Diagnosis of Cancer), please let us know in your cover letter and we will ensure that your paper is treated as if you were responding to this call.

**Reply: Please consider our manuscript for this collection.**

3. Thank you for stating the following in the Competing Interests section: 
   "The CELA3B antibody clone MSVA-410M was provided from MS Validated Antibodies GmbH (owned by a family member of GS)."

Please confirm that this does not alter your adherence to all PLOS ONE policies on sharing data and materials,

**Reply: We have added the statement to the letter (see below) and to the manuscript.**

***“The CELA3B antibody clone MSVA-410M was provided from MS Validated Antibodies GmbH (owned by a family member of GS). This does not alter our adherence to PLOS ONE policies on sharing data and materials.”***

4. In your Data Availability statement, you have not specified where the minimal data set underlying the results described in your manuscript can be found. We will update your Data Availability statement to reflect the information you provide in your cover letter.

**Reply: We have updated our Data Availability statement in the editorial manager:**

***“Raw data are available upon reasonable request. All data relevant to the study are included in the article.”***

5. Your ethics statement should only appear in the Methods section of your manuscript. If your ethics statement is written in any section besides the Methods, please delete it from any other section.

**Reply: We have removed it from the other sections.**

6. Please review your reference list to ensure that it is complete and correct.

**Reply: We have checked our reference list.**

**In reply to the reviewers**

In reply to reviewer #1:

1) To resolve the issue of CELA3B staining in non-acinar pancreatic cells, I suggest the authors use RNA in situ hybridization to look for mRNA expression in these cells.

**Reply: The reviewer recommended adding experimental proof for CELA3B expression in salivary gland cancers. In reply, we have now more prominently highlighted RNA data that support CELA3B RNA expression in various tumor types the introduction on page 3, lines 64-66. We did not perform RNA in-situ hybridization experiments because we made dismal experiences with these technique in tissue microarray spots from formalin-fixed tissues.**

2) The authors should discuss the observation of CELA3B staining in the few salivary gland tumors.

**Reply: We have discussed similarities between salivary glands and the pancreatic gland that might explain occasional CELA3B staining in salivary gland tumors on page 8, lines 187-195.**

3) The quality of the immunostainings in Fig 3 (“non-pancreatic” tumors with positive CELA3B staining) could be improved.

**Reply: The poor quality is only due to the file compression for the review. The original images are high resolution.**

In reply to reviewer #2:

1) Most importantly, the number of positive cases of acinar cell carcinoma of the pancreas, which was only 16, was low not only in the overall database (13,223 cases) but also in comparison. More cases of acinar cell carcinoma of the pancreas are needed in order to draw a conclusion.

**Reply: We agree with the reviewer that more cases would be desirable. However, acinus cell carcinoma is a very rare condition and more cases were not available.**

2-3) How do the authors draw conclusions of high sensitivity (99.9%) of CELA3B immunohistochemistry for diagnosing acinar cell carcinoma of the pancreas? Although CELA3B immunostaining was seen in 12 of 16 (75%) acinar cell carcinoma of the pancreas, but was essentially negative in the remaining 4 cases (20%)，suggesting that the absence of CELA3B immunostaining is a good predictor of acinar cell carcinoma of the pancreas.

**Reply: We now provide detailed data on tumors with and without CELA3B staining in the results section (page 5-6, lines 128-130) that allow for calculating sensitivity and specificity.**

We are grateful for the time and effort taken to review our manuscript.

Sincerely,

Ronald Simon
